# Beeswax by-Products Efficiently Counteract the Oxidative Damage Induced by an Oxidant Agent in Human Dermal Fibroblasts

**DOI:** 10.3390/ijms19092842

**Published:** 2018-09-19

**Authors:** Francesca Giampieri, Massimiliano Gasparrini, Tamara Y. Forbes-Hernández, Piera Pia Manna, Jiaojiao Zhang, Patricia Reboredo-Rodríguez, Danila Cianciosi, Jose L. Quiles, Cristina Torres Fernández-Piñar, Francisco Josè Orantes-Bermejo, Stefano Bompadre, Sadia Afrin, Maurizio Battino

**Affiliations:** 1 Dipartimento di Scienze Cliniche Specialistiche ed Odontostomatologiche (DISCO)-Sez. Biochimica, Facoltà di Medicina, Università Politecnica delle Marche, 60131 Ancona, Italy; f.giampieri@univpm.it (F.G.); m.gasparrini@univpm.it (M.G.); tamara.forbe@gmail.com (T.Y.F.-H.); p.piera@hotmail.it (P.P.M.); zh.jojo@yahoo.com (J.Z.); preboredo@uvigo.es (P.R.-R.); danila.cianciosi@gmail.com (D.C.); 2 Departamento de Química Analítica y Alimentaria, Grupo de Nutrición y Bromatología, Universidade de Vigo, 32004 Ourense, Spain; 3 Department of Physiology, Institute of Nutrition and Food Technology “José Mataix”, Biomedical Research Center, University of Granada, Avda del Conocimiento sn., Armilla, 18100 Granada, Spain; jlquiles@ugr.es; 4 Apinevada Analytical Laboratory of Bee Products, Barrancos s/n, Lanjarón, 18420 Granada, Spain; calidad@apinevada.com (C.T.F.-P.); director@apinevada.com (F.J.O.-B.); 5 Dipartimento di Scienze Biomediche e Sanità Pubblica, Università Politecnica delle Marche, 60131 Ancona, Italy; s.bompadre@univpm.it

**Keywords:** honey, beeswax, ROS, oxidative stress, prevention, antioxidant effects, human dermal fibroblasts

## Abstract

The antioxidant capacity and the phytochemical composition of two by-products from beeswax recycling processes were recently investigated. The aim of the present work was to evaluate the efficacy of one of these by-products, MUD1, against the oxidative stress induced by 2,2′-azobis(2-amidinopropane) dihydrochloride (AAPH) in human dermal fibroblast (HDF) cells. After a preliminary viability assay, the protective effect of MUD1 was investigated through the measurement of apoptosis level, the reactive oxygen species (ROS) and nitrite (NO_2_^−^) production, the level of protein and lipid biomarkers (carbonyl groups, total glutathione and thiobarbituric acid-reactive substance) of oxidative damage, and the measurement of antioxidant enzymes activities (glutatione peroxidase, glutathione reductase, glutathione transferase, superoxide dismutase and catalase). The obtained results showed that MUD1 exerted protective effects on HDF, increasing cell viability and counteracted the oxidative stress promoted by AAPH-treatment, and improved mitochondria functionality and wound healing capacities. This work shows the antioxidant effects exerted by beeswax by-products, demonstrating for the first time their potential against oxidative stress in human dermal fibroblast cells; however, further research will be necessary to evaluate their potentiality for human health by more deeply in vitro and in vivo studies.

## 1. Introduction

The reduction of the energy and water consumption, and in a residual way, the recovery of energy from waste represent the actual main goal of the food industry [1]. For this reason, investment in research, new production systems and recovery technologies for the recycle of food waste biocomponents, are becoming an urgent need [1]. The development of these strategies can lead to numerous advantages, such as the valorization of bioactive molecules that could be used in the food chain or for medicinal purposes. Some examples are represented by collagen derived from fish (e.g., skin, bones, and fins), seaweed or plants [1]. In this context, the products derived from honey production such as beeswax, propolis, pollen, venom and royal jelly are attracting attention of the scientific community, due to their interesting nutritional composition [2,3], which could have a potential impact on biomedicine. In particular, beeswax and its derivatives have been recognized for their antibacterial properties and traditionally used for the treatment of wounds, burns, psoriasis, and topic dermatitis [4]. According to the European food safety authority, beeswax is accepted for its safety and can also be used as a glossing, flavoring, preserving or supplementing agents for food [5]. It is estimated that approximately 30 kg of honey yielded 1 kg of wax, so the use of the latter could have a significant economic impact [3]. In addition, a recent study from our group highlighted the proximal, nutritional, and phenolic content as well as the antioxidant capacity of two beeswax recycling by-products (MUD1 and MUD2). We also demonstrated their anticarcinogenic effects in human hepatocellular carcinoma (HepG2) cells [2]. Both derivatives—although to a greater extent MUD1—induced cytotoxic effects in this cell line by reducing cell viability, increasing reactive oxygen species (ROS) generation, and deteriorating mitochondrial functionality, thanks to the phytochemical contents [2].

Oxidative stress represents the main cause of most common chronic diseases including inflammation, cardiovascular diseases, diabetes, metabolic syndrome, and cancer [6]; in recent years, numerous studies have demonstrated that dietary antioxidants from plant foods represent an efficient strategy to counteract this condition, and can be considered a useful tool for the maintenance of human health status and well-being conditions [7,8,9,10]. On the experience of our previous reports [7,8,9,10] and the antioxidant potential of MUD1 (it presents 1435.66 ± 71.78 mg/100 g and 295.84 ± 14.80 mg/100 g of Total phenolic content and total flavonoids content, respectively) [2], in the present study we evaluated the efficacy of beeswax by-products, MUD1, as a potential therapeutic agent against oxidative damage induced by 2,2′-aszobis(2-amidinopropane) dihydrochloride (AAPH) in Human Dermal Fibroblasts (HDF). HDF are considered an excellent model system to study several aspects of cell physiology, and are widely used to evaluate the in vitro effect of substances of interest in the prevention of oxidative damage caused by different agents [8,11]. We hypothesized that if beeswax byproducts, such as the potent antioxidant MUD1, could protect HDF cells from oxidative damage induced by AAPH, they could be considered as potential therapeutic agents used to speed up wound healing in patients suffering from chronic diseases. To quantify the direct protective effects of MUD1 against ROS damage by AAPH on HDF cells, we measured the cell viability, apoptosis rate, ROS and NO_2_^−^ levels, and the biomarkers of oxidative damage to biomolecules (i.e., proteins and lipids). Moreover, to assess the indirect effects of MUD1 protecting against ROS damage by AAPH on HDF cells, we measured the activity antioxidant enzymes, the mitochondria functionality, and the wound healing capabilities. To the best of our knowledge this is the first study that investigates the protective effect of beeswax by-products on AAPH-induced damage in the HDF cell line.

## 2. Results and Discussion

### 2.1. MUD1 Treatments Reduced AAPH-Mediated ROS Production and NO_2_^−^ Accumulation

**Figure 1:**
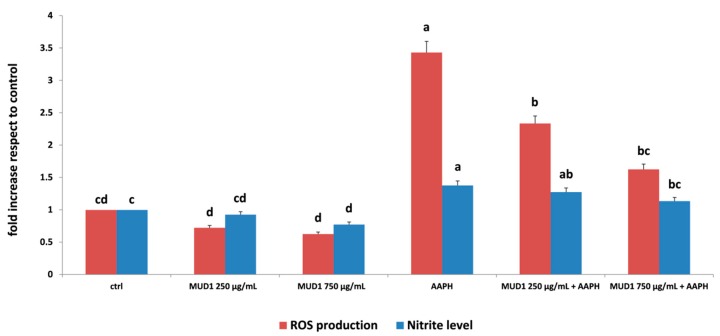
Reactive oxygen species (ROS) production (red bars) and NO_2_^−^ level (blue bars) in human dermal fibroblast (HDF) cells treated with different concentrations of MUD1 (250–750 μg/mL) for 24 h, AAPH (10 mM) for 24 h and with different concentrations of MUD1 and then with AAPH. Data are expressed as mean values ± SD. Columns belonging to the same set of data with different superscript letters are significantly different (*p* < 0.05).

According to our recently published results that showed that MUD1 contains high content in fiber, protein, carbohydrate, polyphenol and flavonoid, and presents antioxidant properties [2], we decided to test the efficacy of MUD1 against AAPH-induced stress in HDF cells. The measurement of intracellular ROS production represents a very useful tool for the evaluation of oxidative stress promoted by AAPH [11,12]. The accumulation of ROS can result in the hyperactivation of the inflammatory response, tissue damage, and oxidative stress phenomena [13]. In the present work, the protective effect of MUD1 on AAPH-induced ROS production was highlighted when applied at 250 µg/mL and 750 µg/mL (Figure 1). In HDF cells, MUD1 treatment reduced ROS production compared to the control group. In cells pre-treated with MUD1 and stressed with AAPH, this reduction became significant (*p* < 0.05) at all MUD1 applied concentrations. Nitric oxide (NO) is widely considered an important regulatory and effector molecule with different biological functions and it represents a fundamental component involved in many physiological and pathophysiological processes [13,14]. As reported in Figure 1, MUD1 was able to reduce the level of NO derivative nitrite production compared to untreated cells (*p* < 0.05). AAPH-treatment significantly increased NO_2_^−^ accumulation (*p* < 0.05), which was efficiently counteracted by MUD1 pre-treatment, restoring also in this case, levels similar to a control group at 750 µg/mL. Similar effects were obtained when Manuka honey was used as a therapeutic agent against AAPH induced oxidative stress in HDF cells by reducing intracellular ROS production and NO_2_^−^ accumulation [11]. Also, pre-treatment with strawberry extracts have been demonstrated to counteract the oxidative damage induced by different chemical and biological agents such as hydrogen peroxide [15], ultraviolet radiations [16], and lipopolysaccharide [7,8]. 

### 2.2. Regulation of Apoptosis Level by MUD1

**Figure 2:**
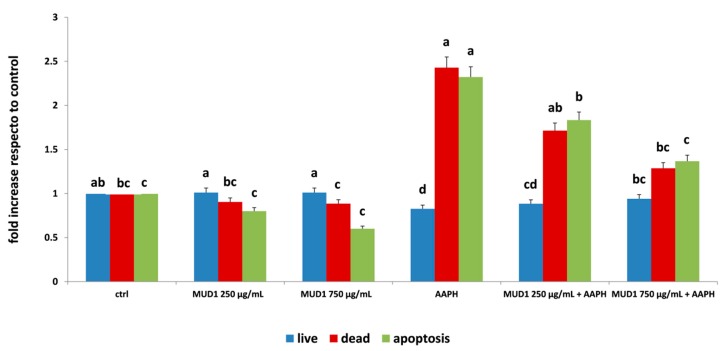
Live (blue bars), dead (red bars), and apoptosis (green bars) levels in HDF cells treated with different concentrations of MUD1 (250–750 μg/mL) for 24 h, AAPH (10 mM) for 24 h and with different concentrations of MUD1 and then with AAPH. Data are expressed as mean values ± SD. Columns belonging to the same set of data with different superscript letters are significantly different (*p* < 0.05).

The augment of ROS production could be related to the induction of apoptosis, a process that leads to many biochemical and morphological modifications, such as cell shrinkage, nucleosomal degradation, chromatin condensation, and the activation of caspases [17]. Our results demonstrated that MUD1 treatment improved HDF viability, increasing the number of live cells and lowering the amount of dead cells at all concentrations applied especially in the presence of APPH (Figure 2). At the same time, AAPH treatment significantly increased the number of apoptotic cells (*p* < 0.05), while MUD1 extracts significantly (*p* < 0.05) reduced this amount at the both concentrations applied. At 750 μg/mL the quantified apoptosis rate was comparable to the untreated group either when applied alone than when applied before incubation with AAPH (Figure 2). These results are similar with those reported for Manuka honey that in HDF significantly (*p* < 0.05) reduced the AAPH induced apoptosis rate for neutralizing the oxidative damage [11].

### 2.3. MUD1 Treatment Reduced Protein and Lipid Biomarkers of Oxidative Stress

In order to quantify the oxidative damage in the HDF cells after treatment with AAPH and/or MUD1, lipid peroxidation and protein carbonyl formation, common markers of lipid and protein oxidation, respectively, were evaluated. Lipid peroxidation is a free radical-mediated chain reaction, which can be stopped through enzymatic means or by free radical scavenging by antioxidants [18]. Some diagnostic tests are available for quantification of the end-products of lipid peroxidation, being the thiobarbituric acid-reactive substance (TBARS) assay the most commonly used. Meanwhile, the production of carbonyl groups represents an early event in oxidative stress, and can be efficiently used to measure the accumulation of protein oxidative damage [19]. In addition to lipid peroxidation and protein carbonyl formation, total glutathione (GSH) level is another important marker of oxidation, since it plays an important role in maintaining the normal reduced state of cells and strongly counteracts the harmful effects of oxidative stress and detoxifying xenobiotics [20].

**Figure 3:**
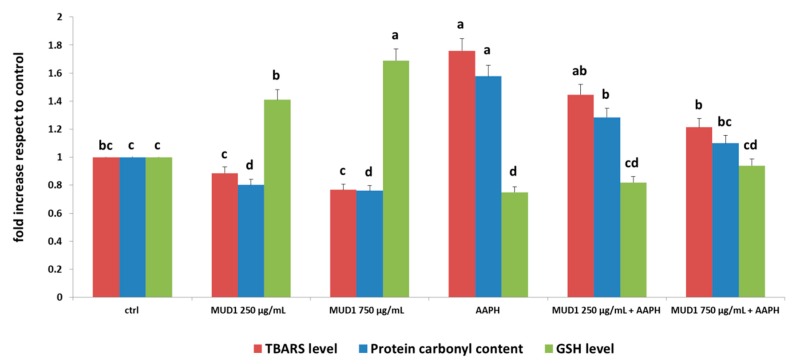
TBARS level (red bars), Protein carbonyl content (blue bars) and GSH (green bars) in HDF cells treated with different concentrations of MUD1 (250–750 μg/mL) for 24 h or AAPH (10 mM) for 24 h and with different concentrations of MUD1 and then with AAPH. Data are expressed as mean values ± SD. Columns with different superscript letters are significantly different (*p* < 0.05).

As reported in Figure 3, MUD1 treatments reduced protein carbonyl content and increased GSH levels compared to untreated cells, while HDF subjected to AAPH treatment showed a remarkable protein damage (*p* < 0.05). Pre-treatment with MUD1 improved the levels of AAPH-induced protein damage, obtaining values similar to the control groups with MUD1 at 750 μg/mL and 250 μg/mL, for GSH and protein carbonyl content, respectively. Similar results were obtained in case of lipid oxidation (Figure 3): MUD1 extract significantly lowered TBARS level in respect to the control group (*p* < 0.05), exerting positive effects also before the oxidative stress induced by AAPH, in when applied at 250 μg/mL and 750 μg/mL (*p* < 0.05). Our findings are in agreement with previous studies reporting the capacity of different natural bioactive compounds or food extract in increasing the levels of GSH in different stressed models [7,8,14,18].

### 2.4. MUD1 Treatment Improved the Endogenous Antioxidant Defence System

**Figure 4:**
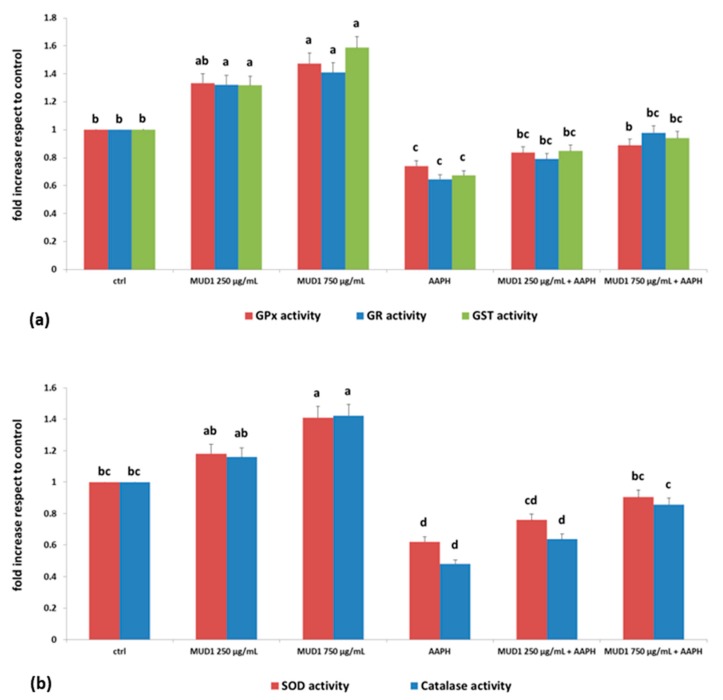
Glutathione peroxidase (GPX), glutathione reductase (GR), glutathione trasferase (GST) (**a**) and superoxide dismutase (SOD) and catalase (**b**) activities cell in HDF cells treated with different concentrations of MUD1 (250–750 μg/mL) for 24 h or AAPH (10 mM) for 24 h and with different concentrations of MUD1 and then with AAPH. Data are expressed as mean values ± SD. Columns with different superscript letters are significantly different (*p* < 0.05).

Different studies indicated that honey and other natural bioactive compounds are able to modulate the activity of diverse antioxidant enzymes, reducing the damage induced by AAPH in HDF cells [11,21]. Under physiological conditions, the balance between prooxidant and antioxidant compounds moderately favors prooxidants, thus inducing a slight oxidative stress, requiring the intervention of endogenous antioxidant systems of the organism [22]. Redox homeostasis of the cell is assured by its complex endogenous antioxidant defense system, which includes endogenous antioxidant enzymes such as superoxide dismutase (SOD), catalase, glutathione peroxidase (GPx), non-enzymatic compounds and low molecular weight scavengers, like uric acid, coenzyme Q, and lipoic acid [23]. The results obtained in our works are in line with these previous data: MUD1 treatment was able to increase the enzymatic activity of GPx, glutathione reductase (GR) and glutathione trasferase (GST) in different doses, obtaining a significant difference in respect to the control group at doses of 250 μg/mL for GR and GST (*p* < 0.05) and at 750 μg/mL for GPx (*p* < 0.05) (Figure 4a). Similar results were obtained for SOD and catalase (Figure 4b). In all the different enzymes, AAPH treatment determined a significant reduction (*p* < 0.05) of enzymatic activities, which were efficiently counteracted by MUD1 treatment in a dose-dependent manner (Figure 4). These results confirm the hypothesis that AAPH causes oxidative damage, decreasing antioxidant enzymes activities since the reservoir is depleted to counteract the damage. Otherwise MUD1 reduced the AAPH effect, improving the enzyme activities and decreasing the induced damage. 

### 2.5. Effect of MUD1 Treatment on OCR and ECAR

**Figure 5:**
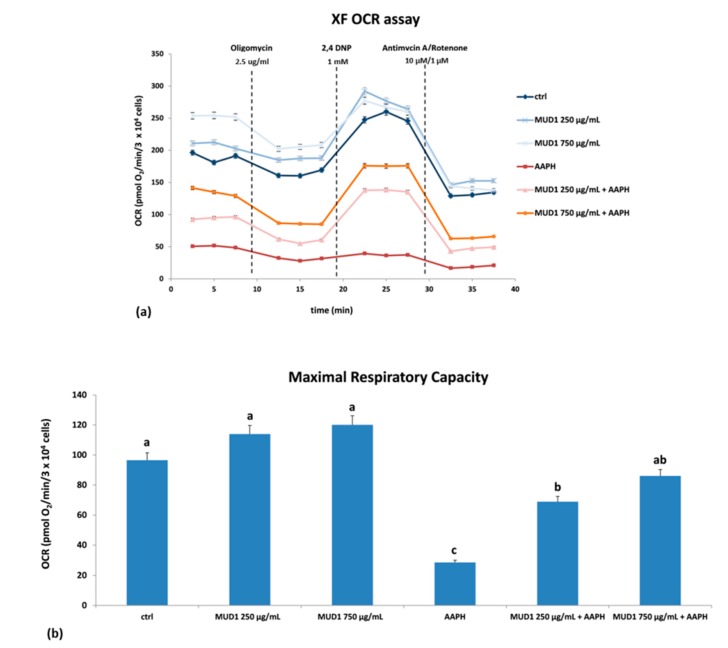
OCR (**a**) and maximal respiratory capacity (**b**) in HDF cells treated with different concentrations of MUD1 (250–750 μg/mL) for 24 h, AAPH (10 mM) for 24 h and with different concentrations of MUD1 and then with AAPH. Data are expressed as mean values ± SD. Columns belonging to the same set of data with different superscript letters are significantly different (*p* < 0.05).

**Figure 6:**
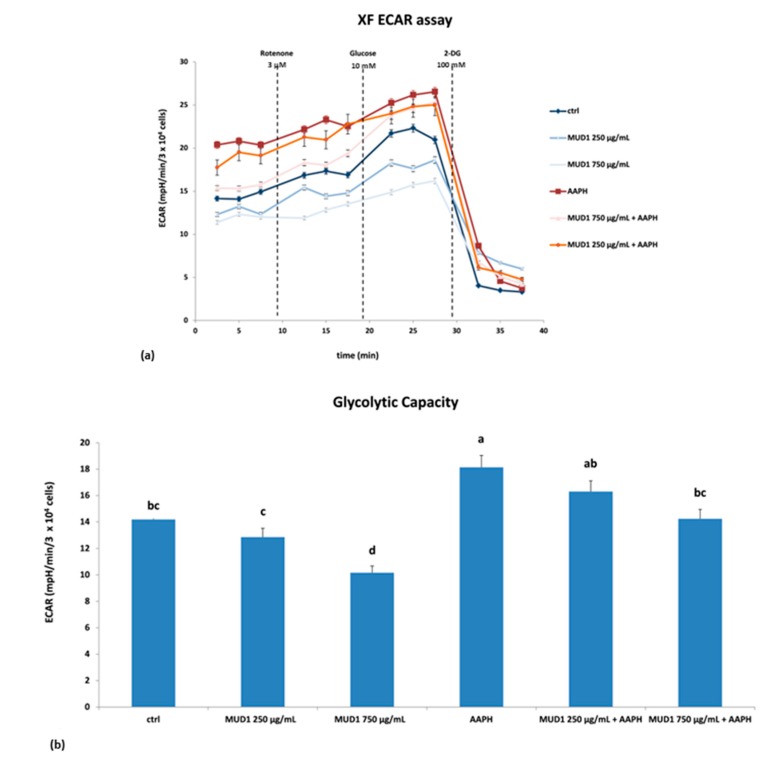
Extracellular acidification rate (ECAR) (**a**) and glycolytic capacity (**b**) in HDF cells treated with different concentrations of MUD1 (250–750 μg/mL) for 24 h, AAPH (10 mM) for 24 h and with different concentrations of MUD1 and then with AAPH. Data are expressed as mean values ± SD. Columns belonging to the same set of data with different superscript letters are significantly different (*p* < 0.05).

The electron transport chain in the mitochondria represents the most important site of ROS production [19]. According to the result found by ROS analysis, we evaluated the mitochondria dysfunction in HDF cells, by assessing oxidative phosphorylation and glycolysis: the first one acts as a major source of adenosine triphosphate (ATP) in almost all aerobic organisms, while glycolysis breaking down glucose produces pyruvate with two ATPs in the cytoplasm [24]. Figure 6a shows the oxygen consumption rate (OCR) trend of the different tested groups, in relation to the molecules applied: oligomycin, an inhibitor of ATP synthase, 2,4-Dinitrophenol (2,4 DNP), an uncoupling agent between the electron transport chain and oxidative phosphorylation, and antimycin A/rotenone, two common inhibitors of electron transport chain. Sequential injections of these compounds calculates basal respiration, maximal respiration, ATP production, proton leak, spare respiratory capacity, and non-mitochondrial respiration. Basal respiration is mainly regulated by the parallel re-entry pathways through the ATP synthase and proton leak. Oligomycin inhibits the ATP synthase and residual respiration is related to the proton leak. The addition of an appropriate concentration of the protonophore 2,4 DNP determines a high artificial proton conductance into the membrane. This maximal respiration is now owned by the electron transport chain activity and/or substrate delivery. The increased respiratory capacity above basal respiration represents the maximal respiratory capacity. In the end, inhibitors of the electron transport chain were injected: antimycin A/rotenone that block complex III and I, respectively. In this way, any residual respiration is non mitochondrial and needs to be removed from the other rates [25]. Starting from the baseline values of OCR, AAPH treatment strongly reduced the oxygen consumption in respect to the control group (Figure 5a). On the contrary, MUD1 treatment improved the mitochondrial respiration, increasing the OCR level in respect to the control and also neutralizing the depressive effect exerted by AAPH. The response to the inhibitor was the same for all the tested groups. Taking into account the maximal respiratory capacity (Figure 5b), MUD1 treatment efficiently improved this value: in particular, pre-treatment with MUD1 at 750 μg/mL before the AAPH incubation significantly contrasted the reduction of maximal respiratory capacity evoked by AAPH (*p* < 0.05). Since the maximal respiratory capacity indicates the maximum rate of respiration that cells can achieve in conditions of high energy demand, an augmentation of this parameter implies that cells are capable of rapidly oxidizing substrates (sugars, fats, amino acids) so that they can face this metabolic challenge. In previous studies our group has shown that other food matrices rich in antioxidant compounds are also capable of stimulating mitochondrial functionality in vitro [11,15] and in vivo [26].

With regard to the glycolytic pathway, starting from the baseline values of the extracellular acidification rate (ECAR), we observed that AAPH treatment remarkably increased the ECAR value, which was reduced through MUD1 treatment (Figure 6a). Furthermore, in this case different molecules were applied: rotenone, glucose, and 2-deoxyglucose (2-DG). As previously indicated rotenone blocks complex I, in this way any effect of mitochondria respiration was averted. The second injection was a saturating concentration of glucose. The cells utilize the glucose injection and catabolize it through the glycolytic pathway to pyruvate, producing ATP, NADH, water, and protons. The extrusion of protons into the surrounding medium causes a rapid increase in ECAR. The final injection was 2-DG, a glucose analog, that inhibits glycolysis through competitive binding to the first enzyme in the glycolytic pathway, glucose hexokinase. The resulting decrease in ECAR confirms that the ECAR produced in the experiment is due to glycolysis, and the difference between ECAR value after glucose injection and the final 2-DG value represent the maximal respiratory capacity [27]. As shown in Figure 6a, the response to the inhibitor was the same for all the tested groups. Finally, looking at the glycolytic capacity (Figure 6b), MUD1 treatment reduced this value significantly at 750 μg/mL (*p* < 0.05). AAPH treatment efficiently raised the value (*p* < 0.05), which was lowered after MUD1 pre-treatment at 750 μg/mL (*p* < 0.05). Our results are in agreement with other published data that showed a significant decrease in ECAR values in lipopolysaccharide (LPS)-treated RAW macrophages after Manuka honey treatment in a dose-dependent way [28], as well as in LPS-treated HDF cells after polyphenol-rich fruits treatment [8].

### 2.6. MUD1 Promotes Tissue Repair by Fibroblast Migration and Wound Closure

Numerous studies have highlighted the capacity of honey in healing chronic wounds in humans and animals [28,29,30,31]. This property can be related to the antibacterial capacity of honey and other mechanisms related to its physical properties, such as pH, and to its immunostimulatory and anti-inflammatory effects [32,33]. Recent studies investigated the properties of honey, in repairing tissue through nitric oxide production, promoting migration, and enhancing wound closure in HDF cells treated with AAPH [11]. On the contrary, in the colon cancer cell model, honey exerted its positive effects reducing cell migration and invasion [27]. The wound scratch assay is a simple, economical, and highly sensitive method to determine cell migration in vitro; it is particularly useful for examining the effects of cell to cell connection and cell to matrix interactions, as well as imitate cell movement during wound healing in vivo. 

**Figure 7:**
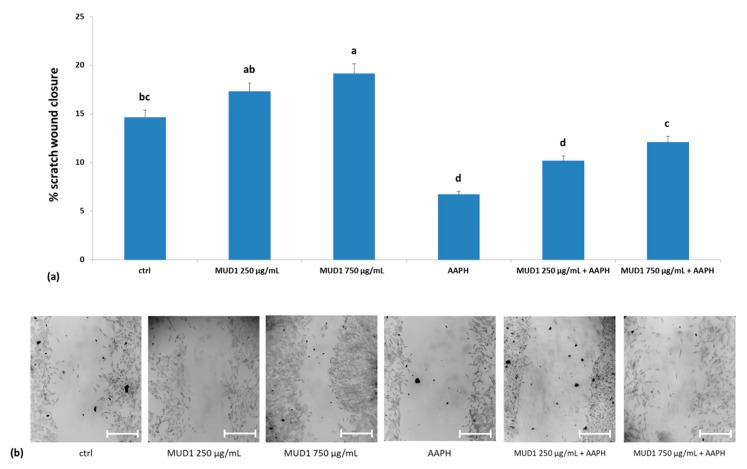
Scratch wound closure (**a**) in HDF cells treated with different concentrations of MUD1 (250–750 μg/mL) for 24 h or AAPH (10 mM) for 24 h and with different concentrations of MUD1 and then with AAPH. Data are expressed as mean values ± SD. Columns with different superscript letters are significantly different (*p* < 0.05). Representative images illustrating the migration of HDF cells into the scratch wound during different treatments exposure (**b**). Scale bars = 100 µm

In the present work a pre-treatment with MUD1 for 24 h slightly promoted scratch wound closure when compared to the control group, at both concentrations (Figure 7a,b). Treatment with AAPH significantly affected (*p* < 0.05) the migration of HDF and wound closure activity, while MUD1 pre-treatment was able to reduce this negative effect, restoring values similar to the control at 750 μg/mL. These results confirm that MUD1 promotes tissue repair by favoring cell migration and it could be used in the topical treatment of wounds, although future in vivo studies would be necessary.

## 3. Materials and Methods

All chemicals and reagents were bought from Sigmae-Aldrich Chemical Company (Sigma-Aldrich, St. Luis, MO, USA).

### 3.1. Sample Collection and Preparation

The combs were collected and subjected to a heating process by steam during the recycling process of the wax honeycombs. A fraction with organic and inorganic waste was isolated from wax (MUD1), which included pollen, molting debris of baby bees, etc. Five samples of MUD1 were randomly harvested from total MUDs and subjected to hydrophilic extraction, as previously reported [34] by diluting 1 g of MUD1 in 10 mL of distilled water and filtered through Minisart filter of 45 μm (PBI International). 

### 3.2. Cell Culture and Treatments

Adult skin HDF were obtained by GIBCO^®^ Invitrogen cell (Waltham, MA, USA), plated into a T-75 flasks and cultured as previously reported [7]. Cells were treated with (i) Dulbecco’s Modified Eagle Medium (DMEM) only (ctrl group); (ii) MUD1 hydrophilic extract for 24 h at 250 and 750 µg/mL; (iii) AAPH for 24 h at 10 mM; (iv) MUD1 for 24 h at 250 and 750 µg/, and then with AAPH 10 mM for 24 h. The combination of dose/time MUD1 treatments was chosen according to our preliminary cytotoxic study. The dose/time treatment of AAPH was chosen according to our previous studies [11].

### 3.3. TALI^®^ ROS Concentration Assay

The ROS intracellular levels determination was performed using the CellROX^®^ Orange reagent (Invitrogen, Life Technologies, Milan, Italy) according to the manufacturer’s instructions, as previously reported by our group [24]. Each treatment was performed in three replicates and the final results were reported as a fold increase compared to the control.

### 3.4. Determination of Nitrite Production

NO_2_^−^ in cell culture media was determined by the Griess method [35]. Each treatment was carried out in three replicates and the final results were expressed as a fold increase in respect to the control.

### 3.5. Apoptosis Detection

Apoptosis was assessed by the Tali™ apoptosis assay kit (Invitrogen™, Life Technologies, Monza, Italy), which uses Annexin V Alexa Fluor™ 488 (Invitrogen™, Life Technologies, Monza, Italy) and propidium iodide (Invitrogen™, Life Technologies, Monza, Italy) to differentiate cells as live, dead, or apoptotic as previously indicated [24]. Each treatment was performed in three replicates and the final results were reported as a fold increase compared to the control.

### 3.6. Measurements of the Protein and Lipid Oxidative Damage

For the measurement of protein and lipid oxidative damage, each HDF was treated as previously reported [36,37,38,39]. Each sample was analyzed in three replicates and the final results were expressed as a fold increase in respect to the control.

### 3.7. Antioxidant Enzyme Activities

HDF cells were incubated with a RIPA buffer on ice for 5 min and the lysate analyzed for the antioxidant enzyme activities of GPx, GR, GST, SOD, and catalase as previously reported by Giampieri et al. [40]. Each treatment was carried out in three replicates and the final results were expressed as a fold increase in respect to the control.

### 3.8. Determination of Mitochondrial Respiration and Extracellular Acidification Rate in Cells

XF-24 Extracellular Flux Analyzer was employed to evaluate in real-time OCR and ECAR, as previously indicated [8,25]. 

### 3.9. In Vitro Skin Fibroblast Migration and Proliferation Assay

HDF cells were seeded into 12-well plate and assessed as previously reported [11,41]. The experiments were made in triplicate and five representative images were made for each wound at randomly chosen points. 

### 3.10. Statistical Analysis

STATISTICA software (Statsoft Inc., Tulsa, OK, USA) were used to perform the statistical analysis. Data were subjected to one-way ANOVA analysis of variance for mean comparison, and significant differences among different treatments were calculated according to HSD Tukey’s multiple range test. Data are reported as mean ± SD. Differences at *p* < 0.05 were considered statistically significant.

## 4. Conclusions

The research of a new life for by-products, before they become waste, is the new goal of the food processing industry. In this study we confirmed and underlined the important antioxidant effects of beeswax by-products, which are mainly related to their phenolic contents, and in particular to flavonoids, as we have previously reported [2]. It is widely accepted that these compounds are able to exert many healthy effects, and the antioxidant capacity is one of the most well-known and studied properties in different experimental models; reported for quercetin and its glycosides, raw honey on Cu^2+^-induced oxidative stress in HepG2 cells [42,43], LPS-stimulated RAW 264.7 cells [44,45], strawberry anthocyanins on AAPH-, and LPS-induced stress on human dermal fibroblasts [8,21]. In the present work, we showed for the first time the protective effect of beeswax by-products against oxidative damage induced by AAPH in HDF cells. An improvement of oxidative status and antioxidant defenses was detected; simultaneously an enhancement of mitochondria functionality and wound healing properties were also marked. The results obtained can represent an interesting starting point for the development of new functional foods based on beeswax by-products. Future studies that investigate the molecular mechanisms involved in these antioxidant properties must be performed, both through in vitro and in vivo models.

## Abbreviations

2,4 DNP	2,4-Dinitrophenol
2-DG	2-deoxyglucose
AAPH	2,2′-azobis(2-amidinopropane) dihydrochloride
ABB	annexin binding buffer
DMEM	Dulbecco’s Modified Eagle Medium
ECAR	extracellular acidification rate
HDF	human dermal fibroblast
HepG2	human hepatocellular carcinoma
GPx	glutathione peroxidase
GR	glutathione reductase
GSH	glutathione
GST	glutathione trasferase
NO	nitric oxide
NO_2_^−^	nitrite
OCR	oxygen consumption rate
PBS	phosphate-buffered saline solution
ROS	reactive oxygen species
SOD	superoxide dismutase
TBARS	thiobarbituric acid-reactive substances
